# LncRNA SNHG5 Suppresses Cell Migration and Invasion of Human Lung Adenocarcinoma via Regulation of Epithelial-Mesenchymal Transition

**DOI:** 10.1155/2023/3335959

**Published:** 2023-01-19

**Authors:** Zhirong Li, Yipeng Wu, Cong Zhang, Suli Dai, Sisi Wei, Ruinian Zhao, Feng Gao, Lianmei Zhao, Baoen Shan

**Affiliations:** ^1^Research Center, The Fourth Hospital of Hebei Medical University, Shijiazhuang, Hebei 050011, China; ^2^Provincial Center for Clinical Laboratories, The Second Hospital of Hebei Medical University, Shijiazhuang, Hebei 050000, China; ^3^Department of Thoracic Surgery, The Fourth Hospital of Hebei Medical University, Shijiazhuang, Hebei 050000, China

## Abstract

Long noncoding RNAs (lncRNAs) are gradually being annotated as important regulators of multiple cellular processes. The goal of our study was to investigate the effects of the lncRNA small nucleolar RNA host gene 5 (SNHG5) in lung adenocarcinoma (LAD) and its underlying mechanisms. The findings revealed a substantial drop in SNHG5 expression in LAD tissues, which correlated with clinical-pathological parameters. Transcriptome sequencing analysis demonstrated that the inhibitory effect of SNHG5 was associated with cell adhesion molecules. Moreover, the expression of SNHG5 was shown to be correlated with epithelial–mesenchymal transition (EMT) markers in western blots and immunofluorescence. SNHG5 also had significant effects of antimigration and anti-invasion on LAD cells *in vitro*. Furthermore, the migration and invasion of A549 cells were suppressed by overexpressed SNHG5 in the EMT progress induced by transforming growth factor *β*1 (TGF-*β*1), and this might be due to the inhibition of the expression of EMT-associated transcription factors involving Snail, SLUG, and ZEB1. In LAD tissues, the expression of SNHG5 exhibited a positive association with E-cadherin protein expression but a negative correlation with N-cadherin and vimentin, according to the results of quantitative real-time PCR (qRT-PCR). In summary, the current work demonstrated that the lncRNA SNHG5 might limit cell migration and invasion of LAD cancer via decreasing the EMT process, indicating that SNHG5 might be used as a target for LAD therapeutic methods.

## 1. Introduction

Lung cancer has become one of the most common malignant tumors, as well as the leading cause of cancer-related death globally. Lung adenocarcinoma (LAD) is the most familiar type of primary lung cancer and is generally diagnosed at an advanced stage [1]. The treatment options available for LAD include surgery, chemotherapy, radiation, and biological therapy [2–4]. Tumorigenesis is an intricate process that involves various genetic alterations and finally leads to malignant transformation [4]. Despite the fact that immune-based approaches and chemoprevention initiatives have revolutionized the treatment of metastatic illnesses, medication toxicity and the growth of resistance remain major difficulties [3–5]. Thus clarifying the process of tumor growth and establishing novel treatment targets is critical for the treatment of cancer. However, the details of the molecular mechanisms promoting LAD progression have yet to be clarified.

Long noncoding RNA (lncRNA) is defined as a transcript with a length of more than 200 nucleotides, which plays a significant role in human cancer through chromatin remodeling, transcription, and post-transcriptional regulation [6]. The 524-bp small nucleolar RNA host gene 5 (SNHG5 or U50HC) belongs to the non-protein-coding multiple small nucleolar RNA (snoRNA) host gene family and the 5′-terminal oligopyrimidine gene class [7]. It was formerly reported that SNHG5 is functionally related to many kinds of tumors, including chronic myeloid leukemia [8], gastric cancer [7, 9], colorectal cancer [10], melanoma [11], and bladder cancer [12]. In addition, a recent document proclaimed that the overexpression of SNHG5 sensitized LAD cells to gefitinib treatment by interacting with miR-377 to modulate its downstream target, CASP1 [5]. However, the functions and molecular basis of SNHG5 in LAD cells and their correlation with LAD pathogenesis have not been further explored.

In the present study, SNHG5 was shown to be significantly downregulated in LAD tissues. *In vitro*, SNHG5 overexpression inhibited lung cancer cell migration and invasion, whereas SNHG5 knockdown increased LAD cell migration and invasion. In addition, our results revealed that SNHG5 plays a role in LAD advancement via modulating the epithelial-mesenchymal transition (EMT) of lung cancer tissues, indicating that SNHG5 might be a valuable target in the treatment strategy for LAD.

## 2. Materials and Methods

### 2.1. Tissue Specimens

The tumor tissues and normal adjacent tissues from 61 patients with LAD were collected from 2014 to 2017 at the Fourth Hospital of Hebei Medical University. The noncancerous tissues were obtained at least 5 cm from the tumor site. Patients who underwent surgical resection and received no preoperative radiotherapy or chemotherapy were enrolled. Two independent pathologists confirmed the histopathological diagnosis according to World Health Organization (WHO) standards. The tissue samples were promptly frozen in liquid nitrogen and preserved at –80°C after surgical excision. The newest UICC (Union for International Cancer Control) and AJCC (American Joint Committee on Cancer) tumor classifications and tumor node metastases (TNM) stages were described. The Fourth Hospital of Hebei Medical University's Ethics Committee accepted this study [Approval # 2019057]. At the beginning of this study, each participant signed a written informed consent form.

### 2.2. Survival Analysis Using RNA-Sequencing Data from the Cancer Genome Atlas (TCGA)

The Level 3 RNA-seq mRNA expression data (HTseq), clinical elements, and survival outcome data of 513 LAD were downloaded from the TCGA dataset (https://portal.gdc.cancer.gov/) (accessed on March 23, 2021) [13, 14]. All patients meeting a clinical endpoint had >30 days of follow-up. Following this, gene expression levels as FPKM (fragments per kilobase of exon per million fragments mapped) were used for further analysis. We transformed the level three HTSeq-FPKM into transcripts per million reads (TPM) for quantification and comparison [15, 16]. The progression-free interval (PFI) refers to the time during which the disease of the participant does not deteriorate during and after treatment [13]. Disease-specific survival (DSS) refers to the period during which patients have not died from a specific disease [13]. Cox regression analysis and the Kaplan-Meier technique were used to perform the survival analysis. Statistical analysis and visualization were performed in R version 3.6.3.

### 2.3. Cell Lines and Reagents

The cell bank of the Chinese Academy of Sciences' Committee on Type Culture Collection (Shanghai, China) provided the human NSCLC cell lines (A549 and H460). In a humidified incubator, the cell lines were cultured at 37°C with 5% CO_2_ in RPMI 1640 medium (Gibco, Grand Island, NY, USA) supplemented with 10% fetal bovine serum (FBS), 100 U/mL penicillin, and 100 g/mL streptomycin (Biological Industries, Beit Haemek, Israel).

### 2.4. Transfection with Oligonucleotides and Plasmids

Shanghai Generay Bio-tech Co. Ltd. constructed the SNHG5 overexpression plasmid (pCDH-CMV-MCS-EF1-GFP-CD511B-SNHG5) and the vector (Shanghai, China). Two short interfering RNAs (siRNA #1 - #2) targeting SNHG5 were purchased from Shanghai Jikai Gene Chemical Technology Co. Ltd. (Shanghai, China). The sequences of the siRNA and negative control are SNHG5#1 sense 5′-GAGGCCAGAUUGUCUUGGATT-3′; SNHG5#1 antisense 5′-UCCAAGACAAUCUGGCCUCTT-3′; SNHG5#2 sense 5′-GCAACGAUUUCUGGCUAGUTT-3′; SNHG5#2 antisense 5′-ACUAGCCAGAAAUCGUUGCTT-3′; negative control (NC) sense 5′-UUCUCCGAACGUGUCACGUTT-3′; negative control (NC) antisense 5′-ACGUGACACGUUCGGAGAATT-3′. NeofectTM was acquired from Beijing Lianlixin BioTech Co. Ltd and utilized as the transfection reagent according to the manufacturer's instructions (Beijing, China).

### 2.5. Quantitative Real-Time PCR (qRT-PCR)

TRIzol reagent was used to extract total RNA from cells and LAD tissues (Invitrogen, Waltham, MA, USA). Using a Reverse Transcription Kit, an equivalent quantity of RNA was reverse transcribed into cDNA (Promega, Madison, WI, USA). The ABI Prism 7900 Sequence Detection System (Applied Biosystems, Foster City, CA, USA) was used to run RT-PCR using the SYBR-Green PCR Master Mix kit (Promega, Madison, WI, USA). The gene expression was measured using the 2^–ΔΔCt^ of each response. The internal control was GAPDH (glyceraldehyde-3-phosphate dehydrogenase). The primer sequences for qRT-PCR are listed in Table 1.

### 2.6. MTS Assay

Cell viability was evaluated using the 3-(4, 5-dimethylthiazol-2-yl)-5-(3-carboxymethoxyphenyl)-2(4-sulfinyl)-2-H-tetrazolium (MTS) assay. For 24, 48, or 72 hours, 2,000 cells per well were planted into 96-well culture plates; 8 *µ*L of MTS solution (Promega, Madison, WI, USA) was added to each well at each time point and incubated at 37°C in the dark. A spectrophotometer was used to measure the absorbance of each well after two hours at 492 nm (Anthos, Salzburg, Austria). Each experiment was carried out three times, with six replications in each group.

### 2.7. Cell Invasion and Migration Analysis

A 24-well Transwell insert (Collaborative Biomedical, Bedford, MA, USA) with an 8 *µ*m pore size, and Matrigel precoating was used to assess cell invasion (BD Biosciences, Bedford, MA, USA). The upper compartment was filled with A549 cells (1 × 10^5^) in 200 *µ*L serum-free media, whereas the bottom chamber was filled with 600 *µ*L of medium containing 10% FBS as a chemotactic agent. After 24 hours of incubation, the nonmigratory or noninvasive cells were removed from the top chambers using cotton swabs, and the filters were rinsed with ice-cold PBS. The cells that reached the lower side of the membrane were fixed with 4% paraformaldehyde, stained with 1% crystal violet, and counted using an inverted fluorescence microscope (magnification: ×100). The migration analysis was carried out in the same way as the invasion assay, with the exception that 1 × 10^5^ cells in serum-free RPMI 1640 medium were introduced to the top compartment without the Matrigel, and a chemoattractant of 10% FBS was utilized. All of the experiments were carried out three times.

### 2.8. Wound Healing Assays

The capacity of cells to migrate was further investigated using a wound-healing assay. The transfected cells were cultured for 24 hours in 6-well plates. After that, a monolayer of cells was scraped using a 20 *µ*L sterilized micropipette tip and cultured for another 24 hours. We observed and recorded the progress of the migration at 0, 24, and 48 hours or other indicated time points after wounding and measured and calculated the distance between the two edges of the scratch.

### 2.9. Western Blot Analysis

The transfected cells were lysed in a lysis buffer, and the protein concentrations were determined using the BCA protein assay kit (Thermo Fisher Scientific, Waltham, MA, USA). Subsequently, the proteins were separated by 12% SDS-PAGE gels and transferred onto polyvinylidene fluoride (PVDF) membranes. Blocking was carried out in 5% nonfat dry milk at room temperature for one hour before incubation with the following antibodies at 4°C overnight: rabbit anti-E-cadherin (Proteintech, Rosemont, IL, USA; 20874-1-AP), N-cadherin (Proteintech, Rosemont, IL, USA; 22018), vimentin (Proteintech, Rosemont, IL, USA; 10366), ZEB-1 (Proteintech, Rosemont, IL, USA; 21544), SLUG (CST, 9585), Snail (CST, 3879), and GAPDH (Proteintech, Rosemont, IL, USA; AP0063). Next, the membranes were washed with TBST three times for five minutes each and incubated with the fluorochrome-labeled secondary anti-rabbit IgG (Odyssey, 926-32211) for one hour at room temperature. The signals were visualized using the Odyssey Infrared Imaging System (LI-COR, Lincoln, NE, USA).

### 2.10. Immunofluorescence Assays

SNHG5 or empty vector-transfected cells were seeded on chambered slides of 24-well culture plates for immunofluorescence labeling, as previously reported [17]. Antibodies against E-cadherin, N-cadherin, vimentin, and secondary (FITC)-conjugated rabbit antibody (ZSFB-Bio, Beijing, China; ZF-0311) were used to stain the cells. The images were taken with an Olympus confocal laser scanning microscope and software from Olympus (Nikon, Tokyo, Japan).

### 2.11. Transcriptome (RNA-Seq) Analysis

Three biological replicates for each pair of SNHG5 or empty vector-transfected cells were performed to detect changes in gene expression after SNHG5 overexpression by transcriptome (RNA-seq) analysis. Briefly, the total RNA was obtained using an RNeasy mini kit (Qiagen, Hilden, Germany). A library with paired ends was produced using the TruSeqTM RNA sample preparation kit, according to the TruSeqTM RNA sample preparation protocol (Illumina, San Diego, CA, USA). Briefly, poly-T oligonucleotides were employed to purify poly-A-containing mRNA molecules using magnetic beads.

After purification, the mRNA was fragmented at 94°C for 8 minutes using divalent cations. Reverse transcriptase and random primers were used to copy the chopped RNA into first-strand cDNA. The second-strand cDNA was then synthesized using DNA polymerase I and RNase H. Afterwards, the cDNA fragments were subjected to an end-repair procedure. The ligation of the adapters and the addition of a single “A” base were completed. To construct the final cDNA library, the products were purified and enriched by PCR. To confirm the insert size and determine the molar concentration, the purified libraries were measured using the Qubit® 2.0 Fluorometer (Life Technologies, Carlsbad, CA, USA) and verified using the Agilent 2100 bioanalyzer (Agilent Technologies, Santa Clara, CA, USA). The cBot constructed the cluster with the library diluted to 10 pM. Finally, the fragments were sequenced on the IlluminaNovaSeq 6000 (Illumina, San Diego, CA, USA). Shanghai Sinomics Corporation was responsible for the development and sequencing of the sequence library. The GEO database was used to store the sequencing data (GSE143766).

All differentially expressed genes were mapped to the Kyoto Encyclopedia of genes and genomes enrichment analysis (KEGG) database and searched for significantly enriched KEGG pathways at *p* < 0.05 level, as indicated by reference [18].

### 2.12. Gene Expression Profiling Interactive Analysis (GEPIA) Analysis

Correlation of SNHG5 with other clinical biomarkers was performed based on Gene Expression Profiling Interactive Analysis (GEPIA) database (http://gepia.cancer-pku.cn/index.html), which is an interactive website based on TCGA and Genotype-Tissue Expression (GTEx)-based project [19, 20]. Spearman's rank correlation analysis was used to determine the correlation coefficient between two genes.

### 2.13. Statistical Analysis

The SPSS (version 21.0) program was used for all statistical analyses (IBM, Armonk, NY, USA). For comparison, one-way analysis of variance, Student's *t*-test, and the Mann–Whitney *U* test were used, as described. Spearman's rank correlation analysis was used to look at the relationship between SNHG5 and EMT markers expression. A *p* value of 0.05 was used for significance determination, and all statistical tests were two-sided.

## 3. Results

### 3.1. The Expression of SNHG5 was Decreased in Lung Adenocarcinoma Tissues and Negatively Correlated with LAD Aggressiveness

**Figure 1:**
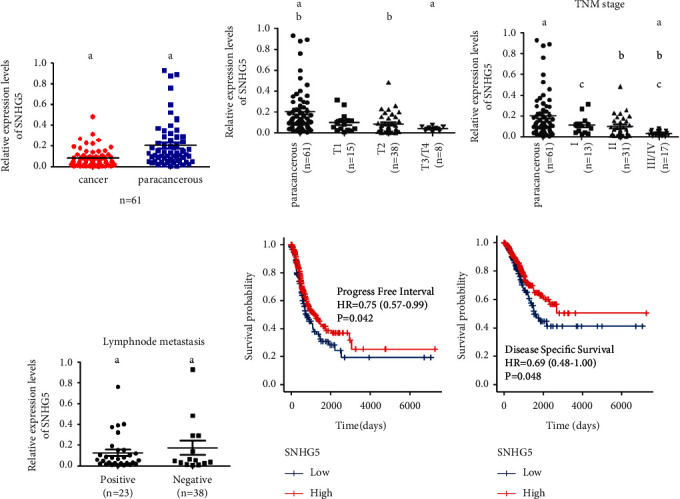
The LncRNA SNHG5 was found to be downregulated in LAD tissues and was associated with the aggressiveness of the disease. (a) The qRT-PCR was used to examine the expression of SNHG5 in 61 samples of LAD tissues and their corresponding neighboring nontumor tissues. The internal control was set to GAPDH. (b) The correlation between SNHG5 expression and size of the tumor. According to the most recent Union for International Cancer Control (UICC) and AJCC (American Joint Committee on Cancer) guidelines, there are four grades of tumor (*T*) that reflect the size of the tumor (area of cancer). (c) The correlation between the expression of SNHG5 and the TNM stages. a: ^*∗∗∗*^*p* < 0.001; b: ^*∗*^*p* < 0.05; c: ^*∗∗*^*p* < 0.01. (d) The association of lymph node metastasis and SNHG5 expression. Negative results indicate no lymph node metastasis, whereas positive results show lymph node metastasis. ^*∗∗*^*p* < 0.01. (e, f) Kaplan-Meier survival curves for progression-free interval, and for disease-specific survival, based on SNHG5 expression data from TCGA. ^*∗*^*p* < 0.05.

The levels of SNHG5 expression in 61 samples of LAD tissues and surrounding normal tissues were first determined using qRT-PCR. As shown in Figure 1(a), the expression of SNHG5 in tumor tissues was much lower than in surrounding normal tissues. Furthermore, the results from controlled clinical trials also revealed that higher expression of SNHG5 is significantly positively correlated with a smaller size of the tumor (Figure 1(b)), earlier TNM stage (Figure 1(c)), and clinical-pathological negative lymph node metastasis (Figure 1(d)) of LAD. What is more, based on RNA-sequencing data of 513 patients from the TCGA dataset, both progression-free interval (PFI) [HR: 0.75 (0.57–0.99), *p* = 0.042] and disease-specific survival (DSS) [HR: 0.69 (0.48–1.00), *p* = 0.048] showed that the higher expression of SNHG5 was significantly associated with longer survival times (Figures 1(e) and 1(f)). These findings showed that abnormalities of SNHG5 expression are important in the development and progression of LAD.

### 3.2. Suppressed Effect of SNHG5 on Lung Cancer Cell Lines Was Associated with EMT

To investigate how SNHG5 affects lung cancer metastasis, we performed transcriptome sequencing to compare the differential expression of genes in the SNHG5 overexpressed lung cancer cell A549 with the control group. The sequencing data were uploaded to the GEO database (GSE143766).

**Figure 2:**
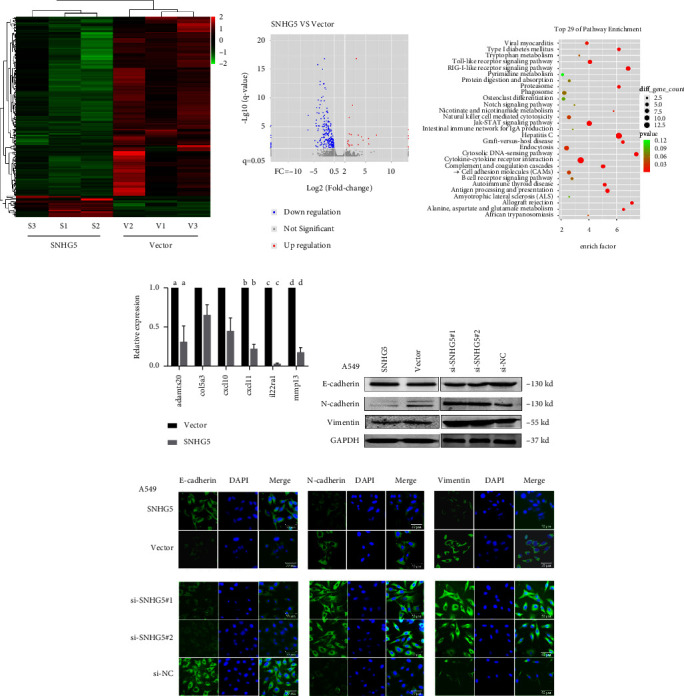
In LAD A549 cells, overexpression of SNHG5 decreases the expression of EMT-associated genes. (a) To evaluate the effect of lncRNA SNHG5 on lung A549 cells, transcriptome sequencing analysis (GSE143766) was performed. According to the color bar in the logarithmic scale given above the heatmap, the red and green colors in the heatmap respectively reflect high and low expression of target genes. (b) Volcano plot analysis of the differential gene expression after SNHG5 overexpression. (c) KEGG analysis of downregulated genes in SNHG5-overexpressing A549 cells. (d) The qRT-PCR analyzed the expression of target genes associated with migration and invasion, which were downregulated in the results of the transcriptome sequencing after SNHG5-overexpression. ^*∗*^*p* < 0.05; (e, f) Western blot and immunofluorescence analysis evaluated the expression of EMT markers in control and A549 cancer cells after transfection with SNHG5 or siRNAs. ^*∗*^*p* < 0.05.

There were 262 dysregulated genes associated with SNHG5 overexpression, including 27 genes that were upregulated and 235 genes that were downregulated (fold change > 2, *p* < 0.01), which were found to be overlapping in both groups (Figures 2(a) and 2(b)). The KEGG pathway enrichment analysis revealed that some downregulated genes were cell adhesion molecules (Figure 2(c)). Six target genes related to migration and invasion were chosen for verification by qRT-PCR analysis in order to corroborate the transcriptome sequencing results [21–23]. As shown in Figure 2(d), the levels of expression of ADAMTS20, COL5A3, CXCL10, CXCL11, IL22RA1, and MMP13 were consistent with the results of the transcriptome sequencing. We hypothesized that the effect of SNHG5 on lung cancer cell lines was associated with EMT based on these findings and the fact that EMT has been strongly linked to tumor progression and metastasis [24]. Therefore, western blot and immunofluorescence were further employed to determine the impact of SNHG5 on the EMT process of LAD. The results showed that overexpressed SNHG5 increased the expression of the epithelial marker E-cadherin while decreasing the expression of the mesenchymal markers N-cadherin and vimentin, whereas SNHG5-shRNA caused a decrease in the expression of E-cadherin while increasing the expression of N-cadherin and vimentin (Figures 2(e) and 2(f)). These findings suggested that SNHG5 might prevent lung cancer cell migration and invasion by suppressing the EMT process.

### 3.3. SNHG5 Inhibited Lung Cancer Cell Migration and Invasion *In Vitro*

**Figure 3:**
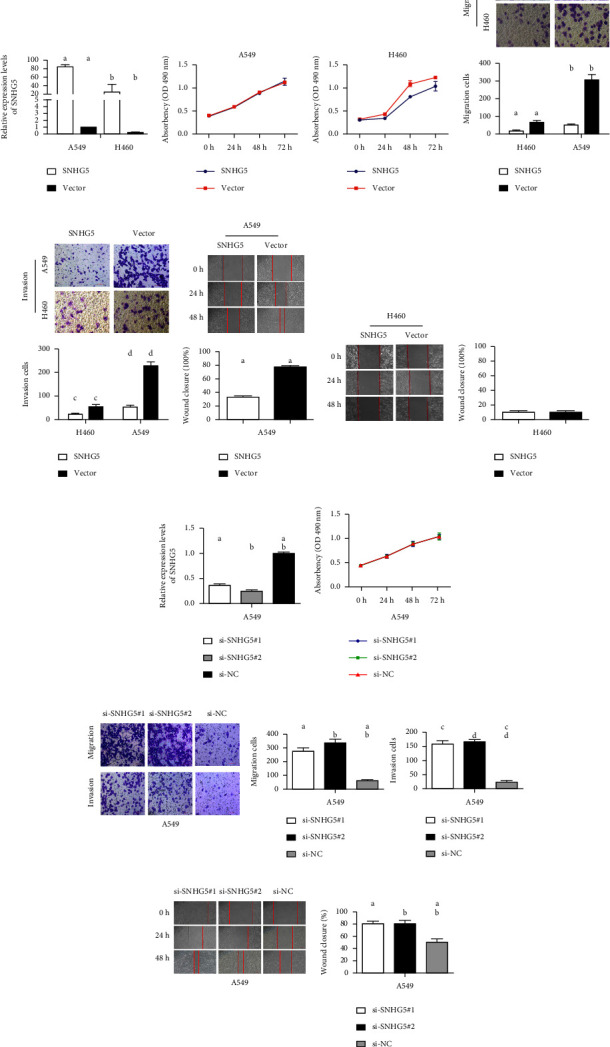
In vitro, SNHG5 inhibits LAD cell migration and invasion. (a) In the controls and LAD cell lines transfected with SNHG5, qRT-PCR analysis of SNHG5 was performed. Statistically, groups identified with various letters differ from one another. *p* < 0.01. (b) The MTS assay was performed in the control and LAD cell lines after transfection with SNHG5, (c, d) Transwell analyses were carried out in normal and SNHG5-overexpressing LAD cell lines; the original magnification was set at ×200. a, c: ^*∗*^*p* < 0.05; b, d: ^*∗∗*^*p* < 0.01. (e, f) Wound healing assay was conducted in the control and SNHG5-overexpressed LAD cell lines. Original magnification, ×100. The groups marked with “a” are statistically distinct. ^*∗*^*p* < 0.05. (g) In the normal and A549 cells transfected with different siRNAs, qRT-PCR analysis of SNHG5 was performed. (h–j) MTS, Transwell, and wound healing analyses were conducted in controls and A549 tumor cells transfected with various siRNAs. a,b: ^*∗*^*p* < 0.05.

Next, in order to evaluate the potential biological role of SNHG5 in LAD, several approaches were used to examine variations in cell growth, invasion, and migration following SNHG5 overexpression or silencing. Initially, the CD511B-SNHG5 plasmid was constructed for the cell experiment. Compared with the empty vector, the expression of SNHG5 increased sharply after transfection with the CD511B-SNHG5 plasmid in both A549 and H460 cells (Figure 3(a)). Furthermore, overexpression of SNHG5 showed little effect on A549 cells but drastically slowed H460 cell growth, according to the MTS assay's growth curves (Figure 3(b)). Additionally, the results of the Transwell tests revealed that the upregulation of SNHG5 markedly decreased the invasion and migration of A549 and H460 in comparison with the control group (Figures 3(c) and 3(d)). Results of wound healing assays showed that the SNHG5 upregulation repressed the migration of A549; however, it had no effect in H460 (Figures 3(e) and 3(f)). Therefore, we selected A549 cells for further investigation of the effects of SNHG5 downregulation.

Two specific siRNAs (siRNA #1 and #2) were shown to have considerably lowered endogenous SNHG5 expression (Figure 3(g)). Though the downregulation of SNHG5 had no effect on the proliferation of A549 lung cancer cells (Figure 3(h)), inhibiting SNHG5 increased cell migration and invasion when compared with the control (Figure 3(i)). Consistent with these results, the downregulation of SNHG5 increased the migration ability of A549 cancer cells (Figure 3(j)). Taken together, these findings suggest that SNHG5 is important in preventing cell metastasis.

### 3.4. The EMT of A549 Was Induced by Transforming Growth Factor *β*1 (TGF-*β*1)

**Figure 4:**
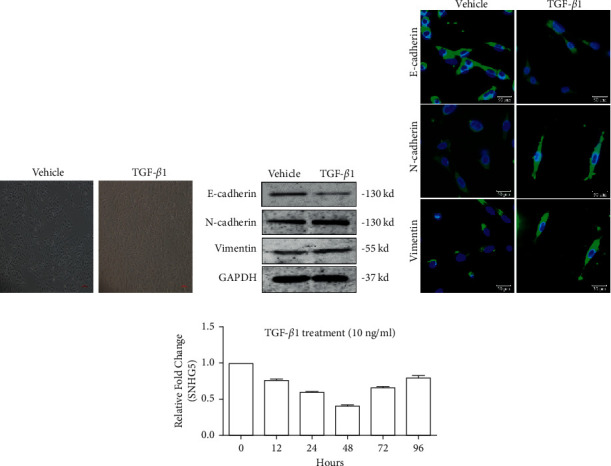
In the EMT of LAD A549 cells caused by TGF*-β*1, the LncRNA SNHG5 was downregulated. (a) An inverted microscope was used to examine morphological alterations in A549 cells generated by vehicle control and 10 ng/mL TGF-*β*1. (b, c) The levels of expression of EMT markers in A549 cells after TGF-*β*1 exposure were investigated using western blot and immunofluorescence. (d) The expression of SNHG5 in A549 cells was investigated using qRT-PCR at various time periods subsequent to TGF-*β*1 exposure.

As well known that TGF-*β*1 is one of the most important extracellular inducers of EMT, which was marked by a reduction in E-cadherin and an increase in N-cadherin [24]. Therefore, to test the influence of SNHG5 on the EMT process of LAD cells, we established an EMT model of A549 cells induced by TGF-*β*1 (10 ng/mL) in the present study. After 48 hours of treatment by TGF-*β*1, A549 cancer cells presented as EMT elongated morphological shapes, while untreated A549 cells still showed oval and epithelial-like morphology (Figure 4(a)). After TGF-*β*1 treatment, the expression of E-cadherin decreased while the expression of N-cadherin and vimentin was increased significantly (Figures 4(b) and 4(c)). Furthermore, the immunofluorescence results further demonstrated that TGF-*β*1 treatment caused considerable alterations in the elongated shape of A549 cells (Figure 4(c)). Herein, TGF-*β*1 was able successfully to induce EMT in A549. At this time, the expression of SNHG5 in TGF-*β*1-induced A549 cells rapidly dropped, reaching its lowest level after 48 hours (Figure 4(d)). Thus, we picked this time period for the ensuing further tests.

### 3.5. The Overexpression of SNHG5 Inhibited the EMT of A549 Cells Induced by TGF-*β*1

**Figure 5:**
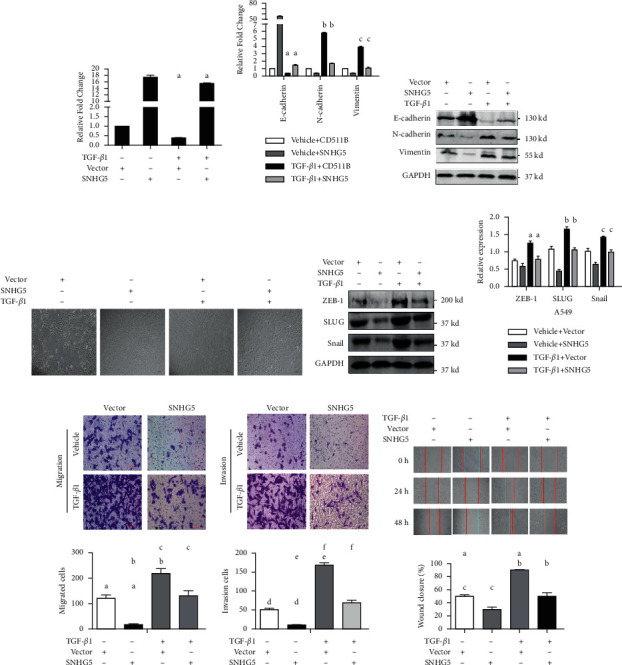
Overexpression of SNHG5 inhibits EMT, preventing LAD cells from migrating and invading. (a) After TGF-*β*1 or vehicle control treatment, qRT-PCR was used to examine the relative fold change of SNHG5 expression in control and SNHG5-overexpressing A549 cells. ^*∗∗∗*^*p* < 0.001. (b) After TGF-*β*1 exposure, qRT–PCR was used to examine the relative fold change of EMT markers in normal and SNHG5-overexpressing A549 cancerous cells. (c) Western blot analysis was used to assess the level of expression of markers in control and SNHG5-overexpressing A549 cancer cells after TGF-*β*1 treatment. (d) The inverted microscope determined the morphological changes in control and SNHG5-overexpressing A549 cells after TGF-*β*1 or vehicle control treatment. (e, f) Western blot and qRT–PCR analysis of EMT-related transcription factors in normal and SNHG5-overexpressing A549 cancer cells following TGF-*β*1 or vehicle control treatment. (g–i) Transwell and wound-healing experiments were conducted on control and SNHG5-overexpressing A549 cancer tissues after TGF-*β*1 treatment. Wound-healing distance was measured and expressed as a percentage of the distance at zero hours. a: ^*∗∗*^*p* < 0.01; b: ^*∗∗*^*p* < 0.01; c: ^*∗∗∗*^*p* < 0.001; d: ^*∗∗*^*p* < 0.01; e: ^*∗∗∗*^*p* < 0.001; f: ^*∗∗∗*^*p* < 0.001.

Next, we overexpressed SNHG5 in A549 cells following TGF-*β*1-induced EMT to elucidate whether it had an influence on the EMT. We first analyzed the SNHG5 expression levels in control and SNHG5-overexpressing A549 cells with or without 48 h of TGF-*β*1 treatment. It was revealed that overexpression of SNHG5 partially blocked the TGF-*β*1-induced reduction of SNHG5 (Figure 5(a)). Furthermore, the overexpression of SNHG5 dramatically reduced the downregulation of E-cadherin and the upregulation of N-cadherin and vimentin produced by TGF-*β*1, as shown in Figures 5(b) and 5(c). Moreover, the overexpression of SNHG5 inhibited TGF-*β*1-induced morphological alterations in A549 cancer cells (Figure 5(d)).

We further performed real-time PCR and western blotting to identify the EMT-related transcription factors in A549 cells, such as Snail, SLUG, and ZEB1, to further investigate the inhibitory impact of SNHG5 on the EMT of A549 cells caused by TGF-*β*1. Accordingly, overexpression of SNHG5 decreased the expression of Snail, SLUG, and ZEB1 in TGF-*β*1 treated A549 cells (Figures 5(e) and 5(f)). Furthermore, overexpressed SNHG5 inhibited the migration and invasion of A549 cells induced by TGF-*β*1 treatment, which was consistent with the change of EMT-related factors (Figures 5(g)–5(i)). These findings showed that overexpression of SNHG5 in A549 cells partly reversed TGF-*β*1-induced EMT.

### 3.6. SNHG5 Reversed the Expression of EMT Markers in LAD Tissues

**Figure 6:**
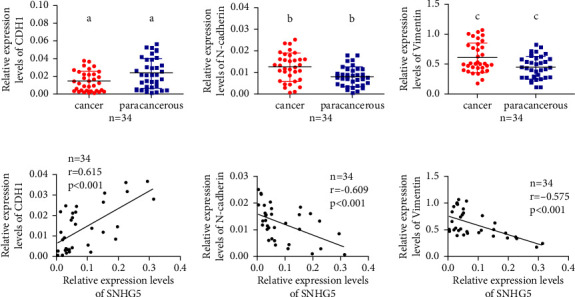
In lung adenocarcinoma tissues, the expression of EMT markers was negatively linked with SNHG5. (a) The expression of CDH1, N-cadherin, and vimentin was examined by qRT-PCR in 34 samples of LAD tissues and their neighboring nontumor tissues. The internal control was set to GAPDH. (b) Pearson's correlation analysis indicated that levels of SNHG5 RNA expression in LAD tissues were positively related to CDH1, but negatively related to both N-cadherin and vimentin ^*∗∗∗*^*p* < 0.001.

To further validate the effect of SNHG5 on EMT of LAD tumor tissues, the levels of expression of EMT markers, including CDH1 (E-cadherin encoding gene), N-cadherin, and vimentin, in LAD and the corresponding noncancerous tissues were measured. Our results showed that, in 34 cases of lung cancer, the CDH1 demonstrated lower expression, while N-cadherin and vimentin had higher levels of expression in tumor tissues, which were significantly different from those in the corresponding noncancerous tissues (*n* = 34, Figure 6(a)). Furthermore, SNHG5 expression was positively linked with CDH1 but negatively with N-cadherin and vimentin (Figure 6(b)). All of these findings indicate that SNHG5 inhibited LAD cancer cell proliferation, migration, and invasion by reversing the EMT process. To further explore the clinical value of SNHG5, we analyzed the correlation of SNGH5 with genes of popular clinical LAD biomarkers, including CEA (CEACAM1, CEACAM3, CEACAM5, CEACAM6), NSE (ENO2), and Mannan-binding lectin-associated serine protease 2 (MASP2) using GEPIA database. The results demonstrated that SNHG5 was negatively correlated with all of these markers (Supplementary Figure 1). Meanwhile, we performed an interaction analysis between popular chemicals and SNHG5 using the Comparative Toxicogenomics Database (CTD) (http://ctdbase.org/) [25]. The result showed that co-treated with Jinfukang (JFK), an effective herbal medicine formula against lung cancer, cisplatin could increase the expression of SNHG5 mRNA in another study (Supplementary Table 1) [26]. Taken together, these results suggested that SNHG5 might have a therapeutic role in LAD suppression.

## 4. Discussion

In the present study, the lncRNA SNHG5 in lung adenocarcinoma cancer cells was identified, and its expression was found to be downregulated in lung cancer cells with respect to tumor size, lymph node metastasis, and TNM stage. A succession of experiments has shown a relationship between lncRNA SNHG5 and EMT biomarkers, leading to the conclusion that lncRNA SNHG5 reduces LAD cell invasion and metastasis by controlling the EMT process. The current findings emphasize the significance of the lncRNA SNHG5 in the development of LAD tumors.

It has been demonstrated by several studies that some lncRNAs, including SBF2-AS1, H19, and OR3A4, have a wide range of applications in the diagnosis and treatment of lung cancer [17, 27, 28]. SNHG5 belongs to a vast family of noncoding genes that host small RNAs such as snoRNAs and microRNAs, which are usually found in the host genes' introns. SNHG5 is known to be highly downregulated in LAD patients with acquired gefitinib resistance, despite the fact that it is infrequently explored in lung cancer [5]. In the current study, the tumor tissues were all isolated from LAD patients without preoperative radiotherapy or chemotherapy. The expression of SNHG5 in adjacent tissues was much greater than that in LAD tissues, as indicated in this study, and downregulation of SNHG5 might increase metastasis and migration in LAD cells. The transcriptome sequencing analysis demonstrated that SNHG5 functioned as a wide gene suppressor in LAD cells. Some of the suppressed molecules were involved with cell adhesion, including mmp13. Matrix metalloproteinases (MMPs) are a class of enzymes that regulate EMT, which is known to promote metastasis in a variety of cancer cells [29, 30]. Moreover, the overexpression of SNHG5 boosted the expression of the epithelial marker E-cadherin while decreasing the expression of mesenchymal indicators N-cadherin and vimentin, according to immunofluorescence and western blot results. Transwell and wound healing assays further revealed that the overexpressed SNHG5 could repress the metastasis and migration of LAD cells in vitro. These results indicate that SNHG5 inhibits EMT, thus reducing lung cancer migration and invasion.

TGF-*β*1 has been shown to promote EMT [31, 32]. We also analyzed the role of SNHG5 in TGF-*β*1-induced EMT in lung cancer cells. In the EMT of cancer tissues, it was found that overexpressed SNHG5 increased the expression of epithelial-related marker E-cadherin while decreasing the expression of mesenchymal-related markers vimentin and N-cadherin, as well as transcription factors. Interestingly, although SNHG5 overexpression had no effect on A549 cell proliferation, as previously identified, it significantly suppressed A549 cell proliferation caused by TGF-*β*1, which was consistent with the relationship between the level of SNHG5 expression and lung cancer tumor size (Figure 1(b)). Therefore, it was assumed that the inhibitory effect of SNHG5 was more likely to act on the typical EMT process but not on the general state of LAD cells. In addition, upregulation of SNHG5 reduced the invasion and migration of H460 cells in Transwell assays, whereas it had no effect in wound healing assays, which indicates that SNHG5 exerts its effect on H460 cells mainly through the regulation of single cell migration rather than collective cell migration. The mechanism of action of SNHG5 on H460 cells needs to be explored further. These results further confirmed that the overexpression of SNHG5 suppressed the lung cancer cell EMT that occurred at the transcriptional level.

SNHG5 has been shown to increase cell migration and invasion in hepatocellular and oral squamous cell carcinomas, whereas we demonstrated the reverse impact of SNHG5 in LAD. Despite the fact that Transwell and wound healing studies *in vitro* demonstrated that SNHG5 inhibited migration and invasion of LAD cells, MTS testing revealed no difference in A549 cell proliferation. Therefore, additional research is needed to understand the essential functions of SNHG5 in human cancer [33]. However, the negative correlation between SNHG5 and several valuable prognostic biomarkers for LAD, including CEA, NSE, and MASP2, and the increased expression of SNHG5 mRNA induced by cisplatin in the previous strongly suggest that SNHG5 might have a therapeutic role in LAD [26]. Thus, to make the current findings more accurate and reliable, more therapeutic information related to SNHG5 should have been obtained for the LAD samples. In the future, as technology progresses and research advances, SNHG5 will aid in the diagnosis and treatment of LAD.

## 5. Conclusions

In conclusion, the current findings have provided a better knowledge of the functions of lncRNAs in LAD etiology and development, suggesting that SNHG5 might have a therapeutic role in LAD.

## Figures and Tables

**Table 1:** Primers for qRT-PCR.

Primer	Primer sequence (5′-3′)
SNHG5 forward	TGGTAGGAACAATGGCGCTG
SNHG5 reverse	TGGCACTAGCCAGAAATCGTT
CDH1 forward	GAAAGCGGCTGATACTGACC
CDH1 reverse	CGTACATGTCAGCCGCTTC
N-cadherin forward	TGTTTGACTATGAAGGCAGTGG
N-cadherin reverse	TCAGTCATCACCTCCACCAT
Vimentin forward	GAGAACTTTGCCGTTGAAGC
Vimentin reverse	TCCAGCAGCTTCCTGTAGGT
CXCL10 forward	CTGAATCCAGAATCGAAGGCCATC
CXCL10 reverse	TGTAGGGAAGTGATGGGAGAGG
MMP13 forward	TGGCATTGCTGACATCATGA
MMP13 reverse	GCCAGAGGGCCCATCAA
CXCL11 forward	GAGGACGCTGTCTTTGCATAGG
CXCL11 reverse	AGCCTTGCTTGCTTCGATTTGG
COL5A3 forward	AACAAGGAAATTTGGACCTCAAGTCC
COL5A3 reverse	TTTGGAGCTGGAGTCTCTGTCTTG
IL22RA1 forward	TGGAGCAGCCCACAGAACTGGA
IL22RA1 reverse	CCCTCAGGACTCCCACTGCA
ADAMTS20 forward	CATACCTACAGCAACATGAATGAAGATC
ADAMTS20 reverse	GAATGTCTTCTTTCATCTTTCAATGGTAC
GAPDH forward	CGGATTTGGTCGTATTGGG
GAPDH reverse	TGCTGGAAGATGGTGATGGGATT

## Data Availability

The datasets used and/or analyzed during the current study are available from the corresponding author upon reasonable request.

## Supplementary Materials

Supplementary Materials Supplementary table 1. Interaction between Cisplatin and SNHG5. Supplementary figure 1. Correlation between SNHG5 and popular LAD clinical biomarkers. A P-value of 0.05 was used for significance determination.
