# Supplementary material for: LncRNA SNHG5 Suppresses Cell Migration and Invasion of Human Lung Adenocarcinoma via Regulation of Epithelial-Mesenchymal Transition
Source: J Oncol. 2023 Jan 19;2023:3335959. doi: 10.1155/2023/3335959 (PMC9879674; doi:10.1155/2023/3335959)
Supplement: Supplementary Materials — Supplementary table 1. Interaction between Cisplatin and SNHG5. Supplementary figure 1. Correlation between SNHG5 and popular LAD clinical biomarkers. A P-value of 0.05 was used for significance determination. [file 3335959.f1.zip › Supplementary table 1.doc]

**Supplementary table 1. Interaction between Cisplatin and SNHG5.**

| **Chemical Name** | **Chemical ID** | **CAS RN** | **Gene Symbol** | **Gene ID** | **Interaction** | **Interaction Actions** |
| --- | --- | --- | --- | --- | --- | --- |
| Cisplatin | D002945 | 15663-27-1 | SNHG5 | 387066 | [Cisplatin co-treated with jinfukang] results in increased expression of SNHG5 mRNA | Affects co-treatment \| increases expression |
